# A crisis of protection and safe passage: violence experienced by migrants/refugees travelling along the Western Balkan corridor to Northern Europe

**DOI:** 10.1186/s13031-017-0107-z

**Published:** 2017-04-16

**Authors:** Jovana Arsenijević, Erin Schillberg, Aurelie Ponthieu, Lucio Malvisi, Waeil A. Elrahman Ahmed, Stefano Argenziano, Federica Zamatto, Simon Burroughs, Natalie Severy, Christophe Hebting, Brice de Vingne, Anthony D. Harries, Rony Zachariah

**Affiliations:** 1 Medecins Sans Frontières, Lekari Bez Granica Strahinica Ban, Belgrade, Serbia; 2 Médecins Sans Frontières, Operational Research Unit (LuxOR) MSF Brussels Operational Center, Luxembourg, Luxembourg; 3 Médecins Sans Frontières, Analysis and Advocacy Unit, MSF Brussels Operational Center, Brussels, Belgium; 4 Operations and Medical Departments, Médecins Sans Frontières, Brussels Operational Centre, Rome, Italy; 5 Operations and Medical Departments, Médecins Sans Frontières, Brussels Operational Centre, Brussels, Belgium; 6 Center for Operational Research, International Union Against TB and Lung Disease, Paris, France; 7 London School of Hygiene and Tropical Medicine, London, UK

**Keywords:** Asylum, Mental health, Traumatic events, Operational Research, MSF

## Abstract

**Background:**

Pushed by ongoing conflicts and pulled by the desire for a better life, over one million migrants/refugees transited Balkan countries and arrived in Europe during 2015 and early 2016. To curb this influx, European countries instituted restrictive migration policies often characterized by building of razor-wire border fences and border closures. Among migrants/refugees who received mental health care in Serbia while travelling through Balkan countries to Northern Europe, we assessed the prevalence and patterns of violent events experienced including physical trauma.

**Methods:**

A mixed methods study among migrants/refugees attending mobile mental health clinics run by Médecins sans Frontières (MSF) between July 2015 and June 2016, in Serbia – a main transit hub to European countries. Clinics were conducted according to MSF guidelines by experienced psychologists who were supported by cultural mediators. The main outcome measures were violent events and associated physical trauma.

**Results:**

Of 992 migrants/refugees attending MSF mental health clinics, the majority (72%) were from Syria and Afghanistan and included vulnerable groups (14%) such as unaccompanied minors and pregnant women. The most frequent mental health symptoms/signs were anxiety (29%) and adjustment reactions (26%). Of the 992 migrants/refugees, 270 (27%) had experienced violent events during their journey. Signs of physical trauma due to acts of violence were seen in 223(22%) of the 992 individuals, 144 (65%) being perpetrated by State authorities and involving women (11%) and children (13%).

Border closures along the Balkan route were associated with a dramatic decrease in registered migrants/refugee arrivals in Serbia. Conversely, among those that made it across the borders, an increasing linear trend in reported violent events was observed at MSF mental health clinics (*X* ^2^ for linear trend, *P* <0 · 001). Qualitative evidence corroborated with quantitative findings.

**Conclusions:**

Nearly one-in-three migrants/refugees seen in MSF clinics experienced violent events including physical trauma along their journey. State authorities, including those in European countries were the perpetrators in over half of such events which were associated with border closures. There is “a crisis of protection and safe passage” which needs to change towards one of respect for the principles of international human rights and refugee law.

## Background

Of recent, Europe has been experiencing one of the most significant influxes of migrants/refugees in its history [1–3]. Pushed by civil wars, terror and pulled by the desire for a better life, people have continued to flee the Middle East, central Asia and Africa, crossing into Europe. During 2015, over one million people arrived by sea to Europe. The main push factors have included ongoing conflicts in Syria, Iraq and Afghanistan and poverty, human rights abuses and worsening security in countries like Pakistan, Eritrea, Iran, and Somalia [2].

**Fig. 1:**
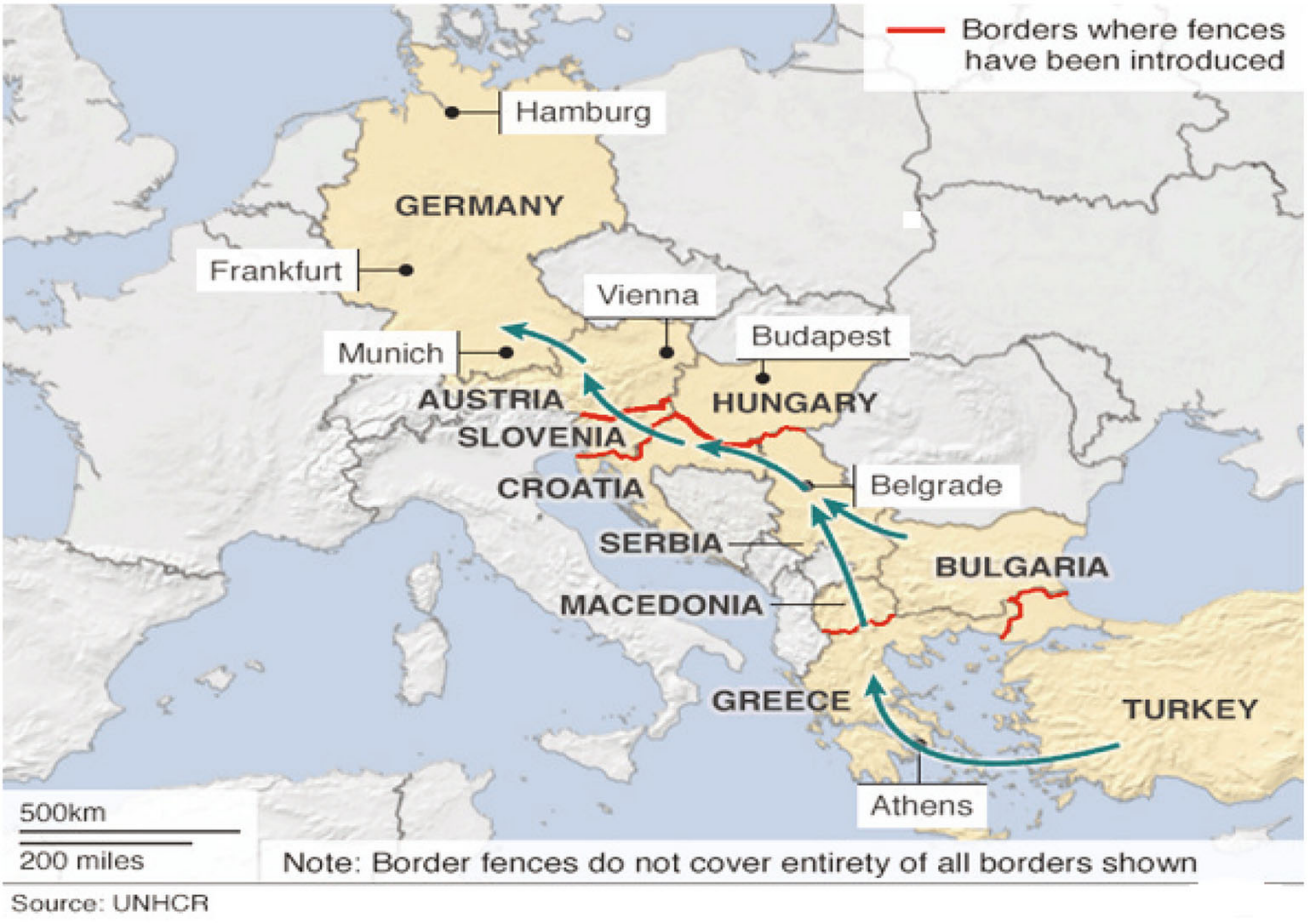
The Balkan route and closures to reduce migration flows to European Countries, 2013–2016

The influx into Europe in 2015 largely occurred through the Balkan corridor. The main branch of this corridor starts in Turkey, passes through Greece into the Former Yugoslav Republic of Macedonia (FYROM) and from there to Serbia, Hungary, Croatia and Slovenia - depending on border closures - and finally into Austria, Germany and beyond (Fig. 1). European countries found themselves unprepared or unwilling to cope with the influx. Despite humanitarian and legal obligations of Europe to treat migrants/refugees with dignity and provide safe havens and asylum [4], what followed was the institution of restrictive migration policies which were often characterized by the building of razor wire border fences and border closures along the Balkan route [5] (Table 1).

**Table 1:** Chronology of Balkan border closures to reduce migrant and refugee flows to Germany and other European countries, 2013–2016

Date	Country	Closure
December 2013	Bulgaria	Builds fence with Turkey making migrants/refugees take the sea route from Turkey to Greece
14th Sept 2015	Austria	Border controls with Hungary
15th September 2015	Hungary	Builds a 175 km fence with Serbia and starts arrestations
16th October 2015	Hungary	Closes border with Croatia forcing people through Slovenia
28th October 2015	Austria	Border fence with Slovenia
11th November	Slovenia	Builds a fence on its border with Croatia
18th November 2015	Slovenia, followed by Croatia, Serbia and Macedonia	Decides to only allow Syrians, Afghans and Iraq national to enter their borders.
February 2016	Macedonia	37 km fence built on the Greek-Macedonian border
February 2016	Countries along the Western Balkan route	Decides to only allow entry on humanitarian grounds to Syrians and Iraqis.

As part of its emergency and humanitarian interventions, Médecins sans Frontierès (MSF) offered mobile mental health clinics for migrants/refugees who transited through Serbia on their journey to European countries. Anecdotal reports from these MSF teams suggested that Balkan border closures were associated with violent events (including intentional physical violence) perpetrated against migrants/refugees [6]. Since MSF clinics routinely record such data [7], there was an opportunity for deeper assessment of the situation. Although a number of studies including a systematic review have assessed the association of potentially traumatic events with depression and Post-Traumatic Stress Disorder (PTSD) [8–11], a PubMed search revealed no studies that focused on violent events encountered by migrants/refugees during their journeys. Getting a better handle on individuals who experience violent events including physical trauma would be useful to guide mental health care. In addition, such information would help assess if the obligation of States “to protect” under International and refugee law is being respected [4] and if not, allow advocacy for corrective measures.

Among migrants/refugees who received mental health care, we thus aimed to assess the prevalence and patterns of traumatic events encountered along their Balkan journey to Europe. Specific objectives were to report on: a) characteristics of individuals attending mental health clinics and their presenting symptoms, b) the pattern of traumatic events including violence, c) physical traumas caused by acts of violence and c) trends in violent events in relation to border closures. In addition, we highlight a few narratives of anonymized testimonies of intentional violence against migrants/refugees.

## Methods

### Design

This was a mixed methods study. The quantitative element involved a retrospective analysis of mental health data recorded by psychologists working in the MSF clinics in Serbia. The qualitative aspect included selected testimonies anonymized) of intentional violence.

### Study Setting

#### General setting

The location of MSF mobile clinics was Serbia, which is situated in the central Balkans, and which borders Hungary to the north; Romania and Bulgaria to the east; FYROM to the south; and Croatia, Bosnia and Montenegro to the west. Serbia’s geographical location makes it a key area as a transit hub for migrants/refugees. Consequent to the growing influx of migrants/refugees, border closures were introduced along the Balkan route (Table 1, Fig. 1). Furthermore, in March 2016, the European Union (EU) and Turkey established a deal to tackle ‘irregular’ migration termed the EU-Turkey deal. Since 20 March 2016, irregular migrants and refugees arriving in Greece are supposed to be systematically sent back to Turkey if they do not apply for asylum or if their claim is declared inadmissible. For each Syrian sent back to Turkey, one Syrian refugee from Turkey was to be resettled in the EU. The EU Turkey deal was accompanied by border closures along the Balkan route for migrants.

#### Specific setting and study sites

MSF teams had been present at key migrant transit locations in Serbia since late 2014 where they offered mobile medical services (including mental health clinics), distribution of non-food items, shelter (tents) and water and sanitation facilities. The MSF strategy in Serbia was to have a flexible and dynamic approach that took into consideration the ever changing migration context. The number of medical teams was thus tailored accordingly, from one team when MSF started working in Serbia, to eight teams at the peak of influx. Mobile teams offered medical and mental health care in the following locations which also corresponded to the study sites:

##### Belgrade

MSF teams were present at the central park and train station. Migrants/refugees typically gathered at these locations during the day to talk to each other, find useful information for the continuation of their journeys, and make plans for travel with smugglers who are usually present. MSF teams also provided care at a reception center for Asylum (the Krnjaca Center for Asylum) which housed registered asylum seekers and those needing accommodation while in transit to other countries.

##### Subotica

MSF teams provided mobile clinic services at two border transit zones, (Kelebjia and Horgos) in this Serbian town bordering Hungary. These zones are entry points into the EU but fenced off with barbed wire fences and manned by armed police and military personnel.

##### Presevo and Sid

These two sites are border entry points into Serbia from Macedonia and into Croatia from Serbia, respectively. Similar to Subotica in the North, MSF clinics in these two location provided primary health care and mental health care for migrants. The period of activities spanned from June 2015 to May 2016.

### MSF mental health clinics and traumatic events

Mental health care is provided in line with MSF guidelines for the implementation of mental health and psychosocial activities in humanitarian contexts [12–14]. A person was considered as having experienced a traumatic event if he/she experienced one or more of a standard list of destabilizing situations (including physical or sexual violence, torture, killings, incarceration) as defined in MSF guidelines [13, 14]. The definitions of traumatic events were developed in-house. A custom designed mobile van was made available for providing mental health consultations. Migrants/refugees were made aware of the existence of the MSF clinics through cultural mediators who conducted group awareness and psycho-education sessions at various gathering points (food access points, parks, sit-outs). These mediators spoke the languages of the migrants/refugees and were from similar cultural backgrounds.

Individuals self-presented to the MSF clinics where care was offered by Serbian psychologists supported by cultural mediators. These mediators are vital to ensure a trans-cultural understanding of mental distress in relation to the social, political, economic, spiritual and cultural views of the beneficiary. Mental health care was focused on three aspects a) *psycho-education* which involves providing information and education on stress reactions and reinforcing positive coping skills, b) *Individual/family psychological support sessions* to support people with moderate and severe mental health conditions/disorders and c) *crisis interventions* involving emergency psychological support after a critical traumatic situation. The intervention facilitated emotional expression (“ventilation”) and stabilization.

Systematic inquiry about traumatic events (including violence) were part of the clinical consultation by the psychologist. Additionally, anyone found with signs of physical trauma was referred to experienced MSF doctors for management. Persons with complicated physical trauma were referred to public hospitals and related costs were covered.

### Study population and period

The study population included all migrants/refugees who presented to MSF clinics and received mental health care in Serbia from July 2015 to June 2016. These migrants were considered “currently on their journey” as they were in transit in Serbia and waiting to travel further into Europe.

### Data collection, variables and data sources

A routine questionnaire for each patient was filled out by psychologists and included socio demographic variables (including age, sex, nationality, vulnerability type) and mental health care information. The latter included types of traumatic event(s), if the event(s) involved violence, type and location of physical trauma (if any), country where the incident(s) took place, perpetrators of violence and categories of mental health symptoms. Psychologists entered these data on a dedicated pro forma which was then transferred into a standardized data base (Microsoft Excel).

For the purpose of this study, traumatic events were classified into violent and non-violent events. A violent event included one or more the following: physical or sexual violence by State authorities or communities, incarceration/kidnapping, family violence, and ill treatment-by State authorities, smugglers or others. All other events were classified as being non-violent. Testimonies of traumatic events and physical violence were collected as part of the routine clerking, transcribed and translated the same day into English and included in the clinical files. Cross-validation of data was done by comparing details in the standardized database with clinical files.

Information on registered arrivals of migrants/refugees to Serbia were sourced from the United Nations High Commissioner for Refugees [3]. This information was used to verify if there was an association between border closures and numbers of arrivals in Serbia.

### Statistical analysis

Trends in violent events seen by month in MSF mental health clinics in Serbia were standardized per 100 mental health consultations. This information along with numbers of migrant/refugee arrivals in Serbia was expressed graphically for the period October 2015 to June 2016. Data on arrivals in Serbia were only available from UNHCR as from October 2015.

Descriptive statistics (numbers, proportions, medians and inter-quartile intervals) were used to report results. Linear trends in violent events (as a proportion of all traumatic events per month) were examined using the chi-square test for linear trend. The level of significance was set at *P* ≤ 0.05 with 95% confidence intervals. Selected testimonies of violent events were reported verbatim after removing any identifiers.

## Results

### Characteristics of individuals attending mental health clinics

The characteristics and referral sources of 992 migrants/refugees who attended MSF mental health consultations are shown in Table 2. The majority (70%) were male, mostly from Syria (46%) and Afghanistan (26%), and individuals had travelled a median of 30 days (IQR 30-120) prior to arrival in Serbia. Vulnerable groups constituted 14% of the total sample with unaccompanied minors, single parents and pregnant women being the most frequent categories. Of 992 individuals who sought mental health care, 828 (83%) had mental health symptoms. The most frequent symptoms were anxiety (29%), adjustment/acute reactions (26%), depression (16%), psychotic disorders(5%), symptoms of Post-Traumatic-Stress-Disorder (PTSD, 5%) and behavioral problems (4%). The main gateway for accessing mental health care was awareness-raising sessions conducted at migrant/refugee sites (Table 2).

**Table 2:** Characteristics of individuals presenting for mental health care, Serbia, July 2015-June 2016 (*n* = 992)

Variable	Number	(%)
Sex		
Female	302	(30)
Male	690	(70)
Age Group in years		
5–17	121	(12)
18–64	856	(86)
≥ 65	15	(2)
Nationality (country of citizenship)		
Syria	454	(46)
Afghanistan	257	(26)
Iraq	90	(9)
Morocco	48	(5)
Iran	41	(4)
Others^a^	102	(10)
Duration of Journey (days) – Median(IQR)	30 (30–120)	
Vulnerability (*n* = 142)		
Unaccompanied minor	41	29
Single parent with a minor	28	20
Pregnant woman	25	18
Disabled	20	14
Known mental Illness	18	13
Elderly >65 years	10	7
Source of referral		
Awareness sessions	761	76
MSF staff	107	11
Friend or family	40	4
NGOs	7	1
Other^b^	67	7
Unknown	10	1

^a^Afghanistan, Greece, Iran, Libya, Montenegro, Syria, Turkey ^b^Health workers, Social workers

### Traumatic events

**Fig. 2:**
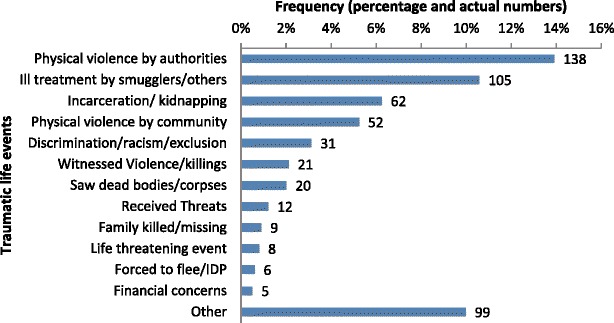
Distribution of of traumatic events, mental health care clinics, Serbia, July 2015-June 2016 (*n* = 992)*

The 992 migrants/refugees experienced a total of 383 traumatic events during their journey from their country of origin, with physical violence being the most frequent (Fig. 2). There were 247 (64%) individuals who had experienced one event, 87(23%) who reported two events and 49 (13%) who reported three or more events.

### Physical traumas caused by acts of violence

Physical trauma due to acts of violence were seen in 223(22%, n-992) migrants/refugees, the majority (65%) being perpetrated by State authorities within or outside Europe and involving women (11%) and children (13%) (Table 3). Among migrants/refugees reporting the location in which they were subjected to physical violence, the most common countries were FYROM, Bulgaria and Hungary.

**Table 3:** Physical traumas related to violence, mental health care clinics, Serbia, July 2015-June 2016 (*n* = 223)

	n(%)
Experienced physical trauma due to violence	223
Sex	
Male	198(89)
Female	25(11)
Age in years	
5–17	29(13)
18–44	181(81)
≥ 45	13(6)
Nationality (country of citizenship)	
Afghanistan	76(34)
Syria	54(24)
Morocco	27(12)
Pakistan	16(7)
Iraq	15(7)
Other	35(16)
Mechanism of injury	
Beating	121(54)
Robbery	44(20)
Beating + Robbery	37(17)
Incarceration	7(3)
Other^a^	14(6)
Country of where the injury took place	
Macedonia	31(14)
Bulgaria	31(14)
Hungary	19(9)
Serbia	11(5)
Other^b^	6(3)
Unknown	125(56)
Perpetrator	
State/Police	144(65)
Community	50(22)
Mafia	26(12)
Other^c^	3(1)

^a^Threatened by a gun or knife, tear gas, rape/forced sex, torture, shot with gun, kidnapped

^b^Afghanistan, Greece, Iran, Libya, Montenegro, Syria, Turkey

^c^Fellow travelers, family members, smugglers

### Violent events in relation to border closures

**Fig. 3:**
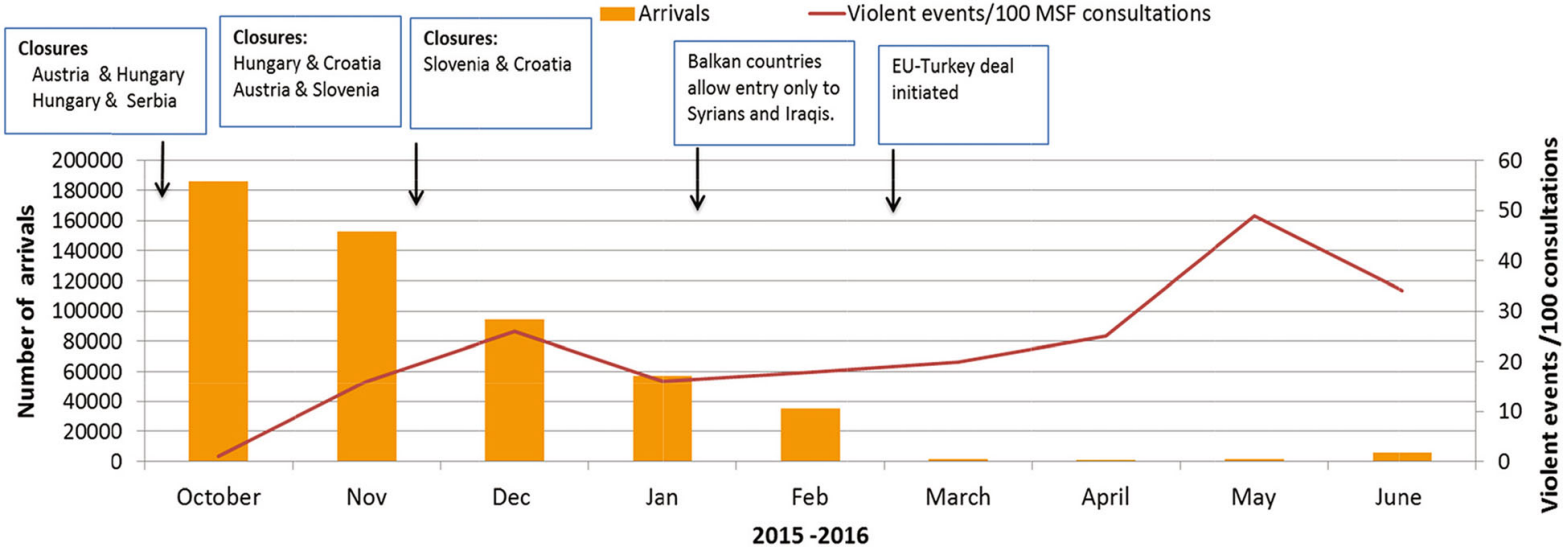
Trend in migrant/refugee arrivals and violent events/100 consultations in Serbia in relation to Balkan border closures (2015–2016)

A total of 270 (27%) individuals out of the 992 migrants/refugees reported having experienced violent events during their journey. Border closures along the Balkan route were associated with a dramatic decrease in numbers of arrivals in Serbia (the main transit hub to Europe) with very few arriving from March to June 2016 (Table 1 and Fig. 3). Conversely, the rate of violent events (per 100 consultations) experienced by those who made it across the borders to Serbia increased in a linear manner over time (chi square for linear trend: 37, *P* <0 · 001).

**Fig. 4:**
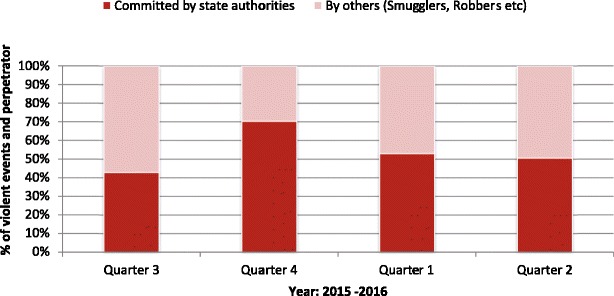
Proportion of violent events perpetrated by State authorities over four quarters, mental health care clinics, Serbia, July 2015-June 2016 (*n* = 270)

Of the 270 violent events reported, over half (*n* = 141, 52%) were perpetrated by State authorities: the proportion rose from 43 to 70% and then decreased and plateaued at 50% during the study period (Fig. 4).

### Case examples of intentional violence perpetrated against migrants/refugees in 2016

Two typical narratives of violent events are illustrated below.

Case example 1: Serbian-Hungarian border – male migrant seen in an MSF clinic

*“I tried to cross the border with a group of migrants. There were five policemen on the Hungarian side who threatened us not to try to cross the border. We lit a fire and sat around about 2 km on the Serbian side. We thought we were safe. The policemen with five big dogs crossed to the Serbian side and chased us. They pushed us on the ground (including women) and kicked us with boots on our face and body. The dogs attacked and bit us too. The police then sprayed pepper spray in our eyes and threw our things into the fire. Some of the women were thrown in the nearby water. One of the policemen was very big and strong and he kept throwing people around. The one that beat me did not stop until I stopped moving and pretended to be dead.”*

Case example 2: Serbian-Hungarian Border– male migrant seen in an MSF clinic

*“I suffered from severe injuries caused by a mine explosion many years ago in Afghanistan that left me physically disabled and with impaired sight. I was travelling with a young relative who was an accompanying aid to me. We had been on the road for about five months. I got treated by MSF in Serbia and received a medical certificate of disability. I was among 10 people who crossed the Hungarian border and were caught by Hungarian police who beat us with their sticks, kicked us with boots also used pepper spray. We were also beaten by civilians.”*

## Discussion

This is one of the first studies assessing traumatic events including violence experienced by vulnerable migrants/refugees who presented for mental health care along the Balkan route to Europe. Border closures in Balkan countries were associated with a considerable decline in arrivals but, conversely, with an increase in violence. Nearly one-in-three migrants/refugees had experienced violent events with State authorities being the perpetrators in over half of such events. The sustained level of violence perpetrated by State authorities (including physical trauma inflicted on women and children) over a considerable period of time (one year of data collection) suggests a “systematic and organized nature” of the violence.

These findings herald serious short-comings in the obligation of Balkan States to provide humane treatment and protection according to the principles and provisions of international and refugee laws [4]. As member States of the United Nations recently gathered in New York for a summit (on 19^th^ September 2016) to agree on a “coordinated and humane approach” to the migrant and refugee issue, these data provide a sobering reality check of the considerable gap between rhetoric and action that needs to be bridged [15].

The study strengths are that recording of traumatic events (including violence) were done according to standardized guidelines, multiple sites were included and data were encoded by trained and experienced psychologists. In addition every psychologist was accompanied by a cultural mediator(s) who spoke the languages of the migrants/refugees and were from similar cultural backgrounds. The use of cultural mediators is crucial as it enhanced dialogue, trust and trans-cultural understanding, and has been recommended as a way to bridge ethno-cultural barriers of communication [9, 16]. We also used a triangulation design for linking quantitative and qualitative information and reporting was in accordance with STROBE guidelines [17].

There are some study limitations. First, data on traumatic events came from interviews conducted through mobile mental health clinics. As many individuals may not have had access to such clinics, we may have under-reported the real situation. Selection bias is very likely. Population based surveys would be the best way of determining the actual prevalence of traumatic events. Second, and understandably, there were variations in the numbers of individuals attending mental health clinics by month. To assess trends, we thus standardized violent events by 100 mental health consultations. Finally, as it is difficult to differentiate between migrants and refugees in transit situations, we considered them as one group “migrants/refugees”. This does not affect the relevance of the study findings as countries should in any case, recognize the human rights of migrants and refugees regardless of legal status.

Notably, this study has a number of policy and practice implications. First, almost four in ten individuals seeking mental health care reported having experienced one or more traumatic events (including violence and traumas) during their journey along the Balkan route. A considerable proportion of such events were perpetrated by authorities (police, border control guards) of European states. This justifies offering mental health care as an integral package of basic medical services at both transit stations and at the destination countries of migrants/refugees. Second, European countries seem to have purposefully introduced a “fortress approach” to blocking entry of migrants/refugees. By so doing, these countries have distanced themselves from their international obligations and have aggravated the predicament of those fleeing hardship. A paradigm shift that fosters a “reception approach” designed to better address the humanitarian and protection needs of vulnerable people is urgently needed.

The lack of alternatives for people to migrate and seek asylum in an organized manner, including policies of *refoulement* [18], push people into the hands of smugglers and make the migratory journeys more dangerous. This sort of treatment also exposes them to kidnappings for ransom, as well as to violence and abuse by organized crime gangs and/or State authorities. We thus call for access to borders and “safe passage” through the swift provision of safe and legal channels for people seeking asylum and for migrants. This may include making wider use of legal entry schemes, such as for example family reunification, humanitarian visas (albeit for restricted time periods), simplified visa requirements, migration pathways, resettlement and relocation.

Finally, the migrants/refugees journey until Serbia lasted between one and four months. This may in part, be explained by the fact that the EU’s response to large movements of migrants/refugees at Europe’s external borders has largely relied on the first country of entry rule [19]. In addition, border closures in Balkan countries might have imposed long stays in the country of first entry and make travel more arduous, lengthy and expensive.

Anecdotal evidence from MSF psychologists suggests that due to long stays and traumatic events, the initial acute reaction of distress experienced by migrants/refugees gradually turns to more complex mental health disorders/psychopathology, which is difficult to manage. Of particular concern are Post Traumatic Stress Disorder (PTSD), major depression and anxiety disorders which have been described in a number of conflict contexts [8, 10, 16]. As MSF mental health services are currently restricted to providing psychological first aid (a short term psychological intervention) and fostering coping mechanisms for acute reactions, ways of expanding the mental health package needs to be considered. Ensuring continuity of care for a dynamic migrant population will also be important. These issues merit further research.

## Conclusion

In conclusion, we have highlighted violent events experienced by migrants/refugees which have been largely perpetrated by State authorities in Europe. In essence there is “a crisis of protection and safe passage” which needs to change towards one of respect for the principles and provisions of international human rights and refugee law.
